# Biological and Genetic Characterization of Pod Pepper Vein Yellows Virus-Associated RNA From *Capsicum frutescens* in Wenshan, China

**DOI:** 10.3389/fmicb.2021.662352

**Published:** 2021-04-15

**Authors:** Jiejun Peng, Shan Bu, Yueyan Yin, Mengying Hua, Kuangjie Zhao, Yuwen Lu, Hongying Zheng, Qionglian Wan, Songbai Zhang, Hairu Chen, Yong Liu, Jianping Chen, Xiaohan Mo, Fei Yan

**Affiliations:** ^1^State Key Laboratory for Managing Biotic and Chemical Threats to the Quality and Safety of Agroproducts, Institute of Plant Virology, Ningbo University, Ningbo, China; ^2^Key Laboratory of Pest Management of Horticultural Crop of Hunan Province, Hunan Plant Protection Institute, Hunan Academy of Agricultural Sciences, Changsha, China; ^3^Longping Branch of Graduate College, Hunan University, Changsha, China; ^4^College of Plant Protection, Yunnan Agricultural University, Kunming, China; ^5^Institute of Alpine Economic Plants, Yunnan Academy of Agricultural Sciences, Lijiang, China; ^6^Yunnan Academy of Tobacco Agricultural Sciences, Kunming, China

**Keywords:** pod pepper vein yellows virus, tombusvirus-like associated RNA, *Polerovirus*, *Umbravirus*, recombination, biological characterization

## Abstract

Tombusvirus-like associated RNAs (tlaRNAs) are positive-sense single-stranded RNAs found in plants co-infected with some viruses of the genus *Polerovirus*. Pod pepper vein yellows virus (PoPeVYV) was recently reported as a new recombinant polerovirus causing interveinal yellowing, stunting, and leaf rolling in *Capsicum frutescens* plants at Wenshan city, Yunnan province, China. The complete genome sequence of its associated RNA has now been determined by next-generation sequencing and reverse transcription (RT) polymerase chain reaction (PCR). PoPeVYV-associated RNA (PoPeVYVaRNA) (GenBank Accession No. MW323470) has 2970 nucleotides and is closely related to other group II tlaRNAs, particularly tobacco bushy top disease-associated RNA (TBTDaRNA, GenBank Accession No. EF529625). In infection experiments on *Nicotiana benthamiana* and *C. frutescens* plants, synergism between PoPeVYVaRNA and PoPeVYV was demonstrated, leading to severe interveinal yellowing of leaves and stunting of plants. The results provide further information on the genetic and biological properties of the various agents associated with pepper vein yellows disease (PeVYD).

## Introduction

Tombusvirus-like associated RNAs (tlaRNAs) are often found in plants infected by some poleroviruses, including those that cause carrot motley dwarf disease, tobacco bushy top disease, and beet western yellows (Sanger et al., 1994; Mo et al., 2011; Campbell et al., 2020; Yoshida, 2020). TlaRNAs are single-stranded positive-sense RNAs of about 3 kb that encode two open reading frames (ORFs): ORF1a ends at an amber stop codon (UAG) and translational readthrough of this codon results in a large protein ORF1b that contains amino acid motifs characteristic of viral polymerases. TlaRNAs lack a coat protein (CP) gene and depend on helper viruses of the genus *Polerovirus* for their encapsidation and transmission. The association of these RNAs with their poleroviruses facilitates movement and increases the accumulation of virus progeny within co-infected cells (Sanger et al., 1994; Syller, 2002; Mo et al., 2015; Yoshida, 2020). Phylogenetic analysis of the full-length genomes of tlaRNAs confirms their relationship to viruses in the genus *Tombusvirus* and that they can be classified into at least two distinct groups (Campbell et al., 2020). The tlaRNAs have a GGL amino acid triplet encoded by the nucleotides immediately following the amber stop codon and eight characteristic motifs of + ssRNA virus RdRps within the deduced amino acid sequences of ORF1b (Koonin, 1991). Notably, the ORF1b of all tlaRNAs has the GDD amino acid triplet characteristic of viral polymerases (Kamer and Argos, 1984).

Pepper vein yellows disease (PeVYD) is a major threat to pepper production in many different countries (Murakami et al., 2011; Dombrovsky et al., 2013; Knierim et al., 2013; Liu et al., 2016; Maina et al., 2016; Lotos et al., 2017). Pepper vein yellows viruses (PeVYVs) induce interveinal yellowing, stunting, and leaf rolling (Kamran et al., 2018). They are phloem-restricted viruses and are currently classified into six species within the genus *Polerovirus* (International Committee on Taxonomy of Viruses [ICTV] 2019 release)^1^, named *Pepper vein yellows virus 1*–*6* (Murakami et al., 2011; Dombrovsky et al., 2013; Knierim et al., 2013; Liu et al., 2016; Maina et al., 2016; Lotos et al., 2017). A PeVYD outbreak on pod pepper (*Capsicum frutescens*) in Wenshan city, Yunnan province in 2019 was associated with a new recombinant polerovirus named pod PeVYV (PoPeVYV) (GenBank Accession No. MT188667). PoPeVYV is predicted to result from a single recombination event with PeVYV-3 as the major parent and the region 4126–5192 nt derived from TVDV as the minor parent. However, a full-length clone of PoPeVYV caused only symptomless infection in *Nicotiana benthamiana* and *C. frutescens* (Zhao et al., 2021).

In this study, we have identified a tlaRNA [PoPeVYV-associated RNA (PoPeVYVaRNA)] associated with PoPeVYV and belonging to Group II of tlaRNAs. This tlaRNA increases the titer of PoPeVYV and has destructive effects on plants. The genomic properties of PoPeVYVaRNA provide insights into the etiological roles of these agents in pod PeVYD (PoPeVYD).

## Materials and Methods

### Sample Collection and RNA Extraction

In July 2019, 89 pepper (*C. frutescens*) samples were collected from three regions of Wenshan city. All the samples had typical viral symptoms of interveinal leaf yellowing and fruit discoloration (Supplementary Figure 1). Total RNA was extracted from fresh leaves/fruits using TRIzol^TM^ Reagent (Invitrogen) in compliance with the manufacturer’s instructions.

### Sequence and *de novo* Assembly

A total amount of 1 μg RNA was used as input material for the RNA sample preparations. The mRNA was purified from total RNA using poly-T oligo-attached magnetic beads. RNA integrity was checked by Agilent 2100 Bioanalyzer (Agilent Technologies). The TruSeq RNA Sample Preparation Kit (Illumina) was used to construct cDNA libraries according to the manufacturer’s instructions.

An Illumina NovaSeq 6000 platform with PE150 bp and CLC Genomics Workbench 20 (QIAGEN) was used for sequencing and data analysis. A total of 6,452,174 paired-end reads were obtained; 78,793 contigs (average length 579 bp) were generated *de novo* and compared with nucleotide and amino acid sequences in GenBank using BLASTn or BLASTx, respectively.

### RT-PCR

Reverse transcription (RT) polymerase chain reaction (PCR) was performed using the ReverTra Ace^TM^ qPCR RT Master Mix (Toyobo) and KOD-plus-Neo (Toyobo) following manufacturer’s protocol. RT was performed with random primers at 42°C for 60 min. The cycling conditions for the subsequent PCR reaction were: 98°C 3 min, and then 30 cycles of 98°C for 30 s, 55°C for 90 s, 68°C for 1 kb/min; and 68°C for 10 min. RT-PCR products were purified, ligated into *pEASY*^®^-Blunt Zero Cloning vector (TRANS, China), and transformed into *Escherichia coli XL10* competent cells, and purified plasmids were sequenced.

### 5′ RACE and 3′ RACE

In order to obtain the full-length sequence of tlaRNA in pod pepper, part of the sequence was amplified using the primer pair tlaRNA-WS (Supplementary Table 1), and specific primers were designed for 5′ RACE R and 3′ RACE F. Then, the 5′ and 3′ RACE reactions were performed to obtain the complete 5′and 3′ terminal sequences. The 5′ and 3′ RACE reactions were performed as previously described (Zhao et al., 2021).

### Phylogenetic and Sequence Analysis

Complete genome sequences of tlaRNAs were obtained from GenBank (Supplementary Table 2). Sequences were aligned using MUSCLE; the evolutionary history was inferred using the maximum likelihood (ML) method (Le and Gascuel, 2008). The best-fit nucleotide substitution model was determined to be ML (GTR + G) by MEGA X (Kumar et al., 2018). Evolutionary analyses were conducted in MEGA X with 1000 bootstrap replicates.

### Plasmid Construction and Agroinfiltration

Reverse transcription PCR was performed using KOD-plus-Neo (Toyobo) and following the manufacturer’s protocol. PCR products were purified with the Gel Extraction Kit (Omega). To generate infectious clones, the ClonExpress II One Step Cloning Kit (Vazyme) was used for homologous recombination. The full length tlaRNA was amplified with primer pair (Inf-tlaRNA) and recombined with the linearized binary vector pCB301-MD (Zhao et al., 2021).

To confirm infectivity, the infectious clone (pCB-PoPeVYVaRNA) was transformed into *Agrobacterium tumefaciens* (GV3101) which was mixed inoculation with pCB-PoPeVYV and delivered to *N. benthamiana* (Zhao et al., 2021). The tissue was harvested 15–28 days post inoculation (dpi). RT-PCR detection was done as described before (Murakami et al., 2011).

### RT-qPCR

Quantitative RT-PCR was used to determine whether the presence of the tlaRNA affects the accumulation levels of PoPeVYV. Fold changes in accumulation of each component were determined using the relative quantification method and normalized to the mean values of those at 28 dpi. For relative quantification of each RNA, the UBC gene of *N. benthamiana* was selected as an internal control.

### Virion Purification

Virions were purified from plants using procedures developed previously (Mo et al., 2010) with some modifications. Virus-infected leaf tissues (250 g) were harvested 28 days post infiltration and homogenized in 500 mL of extraction buffer [0.1 M sodium phosphate buffer; 0.5% (w/v) cellulase; 0.5% (w/v) pectinase; 0.1% (v/v) sodium azide; 0.5% (v/v) β-mercaptoethanol, pH 6.0]. The homogenate was stirred at 25°C for 5 h and emulsified in a mixture of equal volumes of chloroform and 1-butanol. The emulsion was broken by centrifugation at 10,000 *g* for 15 min. Then, Triton X-100 was added to the upper aqueous phase to a final concentration of 1% (v/v) and stirred gently for 30 min. After addition of 8% PEG6000 (w/v) and 0.4 M NaCl, the mixture was stirred gently for 1 h at room temperature, then kept at 4°C for 2 h, and centrifuged at 8000 *g* for 15 min. The resultant pellet was suspended in 50 mL of storage buffer (0.1 M sodium phosphate, pH 7.0) and clarified by centrifugation at 5000 *g* for 15 min. The suspension was concentrated and purified by centrifugation at 70,000 *g* for 4 h through a 30% sucrose cushion. After centrifugation, the pellet was suspended in 1 mL of storage buffer. To exclude any free viral RNA, the virion preparation was digested by RNase at 37°C for 10 min.

### Transmission by Aphids

Virus-free aphids (*Myzus persicae*) were reared from newly born ones. Approximately 100 apterous aphids (3–4 days old nymphs) were transferred using a paintbrush from the virus-free stock plants to a 50 mL centrifuge tube for a starvation period of 1 h. The aphids were then transferred to a cylindrical Perspex cage and allowed to feed on aqueous 20% sucrose solution containing PoPeVYV virions, PoPeVYV + tlaRNA virions or sucrose solution (control). After 24 h, RT-PCR was used to detect virion in aphids (10 per treatment). Then, aphids (30 per treatment) were released onto disease-free pod peppers (six plants per treatment) and kept under controlled environmental conditions (∼25°C, 60% relative humidity, and a 14-h photoperiod). RNA was extracted from the new leaves of these plants after 45 days to test for the presence of viral RNA.

## Results

### Sequence Comparison and Phylogenetic Analysis of PoPeVYV-Associated RNA From Wenshan City

**FIGURE 1:**
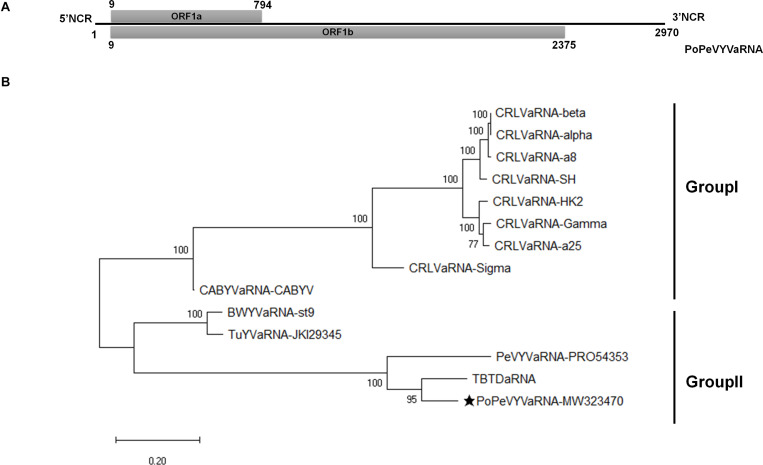
Genome organization and phylogenetic analysis of PoPeVYVaRNA. **(A)** Genome organization of PoPeVYVaRNA. **(B)** Maximum likelihood phylogenetic tree constructed using MEGA X, showing the relationship of PoPeVYVaRNA to other tlaRNAs using their nt sequences. Numbers on branches are bootstrap support values (1000 replicates). Multiple nucleotide sequences were aligned using MUSCLE, the best model (GTR + G), and subsequent analysis was determined by MEGAX. The tlaRNA abbreviations and accession numbers are described in Supplementary Table 2.

Symptoms of interveinal leaf yellowing resembling those caused by viruses were observed in pod pepper fields throughout Wenshan, China and, as we previously reported, a new recombinant polerovirus, PoPeVYV, was identified in 16 of 58 symptomatic samples (Zhao et al., 2021). To assess whether an associated RNA was also present, these leaves were mixed into a pooled sample and sent for next-generation RNA-Seq sequencing (NGS). A large contig of 2915 nt was detected that had the highest nucleotide identity (83.6%) to tobacco bushy top disease-associated RNA (TBTDaRNA; GenBank: EF529625). To confirm our sequencing data, we used primers AR3F and AR5R to amplify a fragment of the tlaRNA by RT-PCR (Campbell et al., 2020). Fragments with a predicted size of approximately 650 bp were obtained in eight of the 16 PoPeVYV-infected samples. Following 5′ and 3′ RACE analysis, we determined the complete sequence of the tlaRNAs, which were identical in sequence and 2970 nt long (GenBank accession number: MW323470). We tentatively designated the isolated RNA as PoPeVYVaRNA (Figure 1A).

The full-length genome sequence of PoPeVYVaRNA had nucleotide identities of 85.5, 76.0, and 65.2% with TBTDaRNA, PeVYVaRNA-PRO54353, and BWYVaRNA-ST9, respectively (Table 1). It had the predicted two ORFs (ORF1a and readthrough protein ORF1b) characteristic of other tlaRNAs, a short 5′ non-coding region of 8 nt preceding the start of ORF1a and a long 3′ non-coding region of 595 nt (Figure 1A).

**TABLE 1:** Comparisons (nucleotide/amino acid identity, %) between the genome of PoPeVYVaRNA and closely related RNAs.

		**5′NCR**	**ORF1^a^**		**ORF1^b^**		**RTD**		**3′NCR**	**Full genome**
		**nt**	**aa**	**nt**	**aa**	**nt**	**aa**	**nt**	**nt**	**nt**
Group II	PoPeVYVaRNA-MW323470	100	100	100	100	100	100	100	100	100
	BWYVaRNA-st9	85.7	49.0	64.0	64.0	67.0	69.5	68.2	57.0	65.2
	TuYVaRNA-JKI29345	85.7	50.5	63.7	63.9	66.6	69.0	67.7	57.3	65.0
	PeVYVaRNA-PRO54353	62.5	63.6	69.7	77.5	76.9	84.2	80.5	73.1	76.0
	TBTDaRNA	**100**	**81.5**	**81.0**	**88.6**	**85.3**	**92.9**	**87.4**	**86.1**	**85.5**
Group I	CRLVaRNA-Sigma	87.5	35.0	58.3	48.9	60.1	54.2	60.9	67.3	59.8

*^*a*^NCR = non-coding region, aa = amino acid, and nt = nucleotide. Highest percentages are underlined and in bold.*

*^*b*^PoPeVYVaRNA (GenBank, MW323470), BWYVaRNA-st9 (GenBank, L04281), PeVYVaRNA-PRO54353 (GenBank, MT321510), TBTDaRNA (GenBank, EF529625), CRLVaRNA-Sigma (GenBank, KM486093), and TuYVaRNA-JKI29345 (GenBank, MK450521).*

To investigate the relationships between PoPeVYVaRNA and other tlaRNAs, the full genome sequences of 13 tlaRNAs were retrieved from GenBank and an ML phylogenetic tree was inferred (Figure 1B). PoPeVYVaRNA clustered into Group II with BWYVaRNA-st9, TuYVaRNA-JKI29345, PeVYVaRNA-PRO54353, and TBTDaRNA (Figure 1B). Alignment analysis also showed that PoPeVYVaRNA was most closely related to TBTDaRNA (Table 1). The predicted amino acid sequence of PoPeVYVaRNA ORF1b contained the eight characteristic motifs of + ssRNA virus RdRps (Supplementary Figure 2) (Koonin, 1991; Campbell et al., 2020).

### PoPeVYV Induces Typical Viral Symptoms in *N. benthamiana* by Co-infection With PoPeVYV-Associated RNA

**FIGURE 2:**
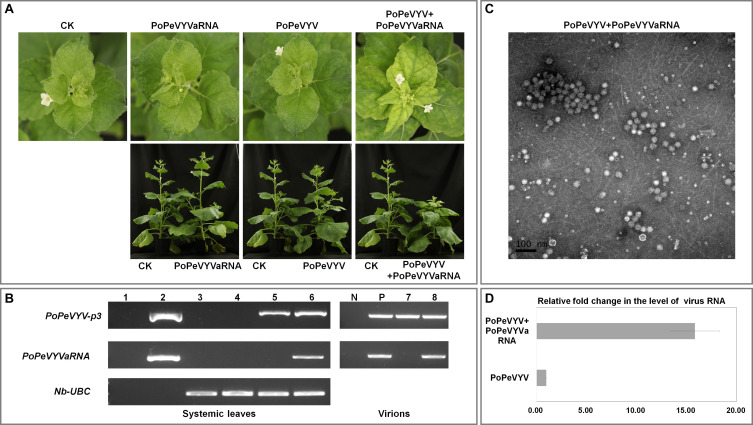
Symptoms caused by PoPeVYV in *N. benthamiana* co-infected with PoPeVYVaRNA. **(A)** Phenotype of *N. benthamiana* plants agroinfiltrated with viral infectious clone combinations or empty agrobacterium (CK) at 28 days post infiltration. **(B)** RT-PCR confirming the presence of viral RNAs in systemic leaves of inoculated plants and virions. 1, negative control; 2, positive control; 3, CK; 4, PoPeVYVaRNA; 5, PoPeVYV; 6, PoPeVYV + PoPeVYVaRNA; N, negative control; P, positive control; 7, virion from PoPeVYV-infected plant; 8, virion from PoPeVYV + PoPeVYVaRNA infected plant. **(C)** Virions purified from leaves infected by the PoPeVYV + PoPeVYVaRNA infectious clones and observed by TEM. Bars represent 100 nm. **(D)** Relative fold changes of PoPeVYV in systemically infected leaves of *N. benthamiana* inoculated with PoPeVYV or PoPeVYV + PoPeVYVaRNA, as shown by quantitative real-time reverse transcription PCR. The means (±*SE*) were calculated from the RNA levels of 12 individual plants at 28 days post inoculation.

To examine the effects of PoPeVYVaRNA on the symptoms caused in mixed infections with PoPeVYV, *N. benthamiana* plants were inoculated by infiltrating their leaves with *A. tumefaciens* harboring different virus–RNA combinations: SI, singly infected with the virus PoPeVYV; SIa, singly infected with PoPeVYVaRNA; MI, mixed infection of PoPeVYV + PoPeVYVaRNA; CK, control with no virus or viral RNA. There were no symptoms in any SI, SIa, or CK plants 14 dpi, whereas at 28 dpi, MI-inoculated plants had typical viral symptoms of interveinal leaf yellowing and plants were stunted (Figure 2A).

Reverse transcription PCR using primer pair (qPoPeVYV-P3) (Supplementary Table 1) to detect the PoPeVYV in systemic leaves showed that viral RNA was present and had spread systemically in SI- and MI-inoculated plants, but not in the controls or SIa-inoculated plants. RT-PCR also showed that PoPeVYVaRNA had spread systemically in MI-inoculated plants and that it was encapsidated in virions purified from MI plants (Figure 2B and Table 2). Quantitative RT-PCR indicated that the level of PoPeVYV RNA in plants inoculated with MI was more than 15-fold that in plants inoculated with SI (Figures 2C,D).

**TABLE 2:** Systemic infection of two host plant species following agroinoculation or aphid transmission with different virus–RNA combinations.

**Virus–RNA combinations**	***Nicotiana benthamiana^1^***	***Myzus persicae^2^***	***Capsicum frutescens^3^***
PoPeVYV	12/12	10/10	2/6
	12/12	10/10	1/6
	12/12	10/10	3/6
PoPeVYVaRNA	0/12	–	–
	0/12	–	–
	0/12	–	–
PoPeVYV + PoPeVYVaRNA	12/12	10/10	5/6
	12/12	10/10	4/6
	12/12	10/10	6/6

*^1^Plants were tested by RT-PCR at 28 days post inoculation.*

*^2^Aphids were tested by RT-PCR at 12 h post virus acquisition.*

*^3^Plants were tested by RT-PCR at 45 days post inoculation.*

**FIGURE 3:**
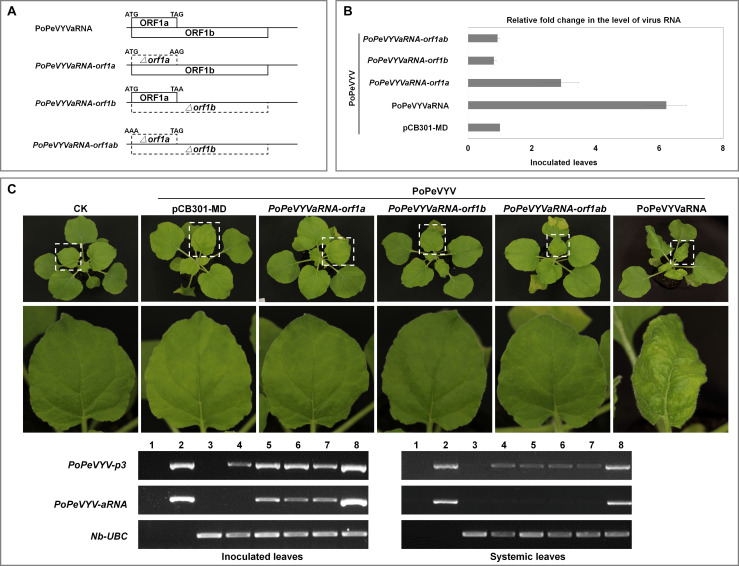
PoPeVYVaRNA is responsible for the synergism with PoPeVYV in *N. benthamiana*. **(A)** The PoPeVYVaRNA mutant infectious clones prepared. **(B)** Relative fold changes of PoPeVYV in local leaves of *N. benthamiana* co-inoculated with PoPeVYVaRNA or mutants of PoPeVYVaRNA (pCB301-MD as control), as shown by quantitative real-time reverse transcription PCR. The means (±*SE*) were calculated from the RNA levels of six individual plants at 3 days post inoculation. **(C)** Phenotype of *N. benthamiana* plants agroinfiltrated with viral infectious clone combinations or empty agrobacterium (CK) at 14 days post infiltration. RT-PCR confirming the presence of viral RNAs in local/systemic leaves of inoculated plants. 1, negative control; 2, positive control; 3, CK; 4, PoPeVYV + pCB301-MD; 5, PoPeVYV + *PoPeVYVaRNA-orf1a*; 6, PoPeVYV + *PoPeVYVaRNA-orf1b*; 7, PoPeVYV + *PoPeVYVaRNA-orf1ab*; 8, PoPeVYV + PoPeVYVaRNA.

To investigate the mechanism of the synergism, mutant infectious clones of PoPeVYVaRNA were constructed that abolished expression of one or both ORFs (*PoPeVYVaRNA-orf1a*, *-orf1b*, and *-orf1ab*). These were used in co-infection experiments with PoPeVYV (six plants per treatment, pCB301-MD as control) (Figure 3A). Quantitative RT-PCR indicated that the level of PoPeVYV RNA in local leaves co-inoculated with PoPeVYVaRNA or *PoPeVYVaRNA-orf1a* was more than 6- or 2.9-fold that in leaves co-inoculated with pCB301-MD. Co-inoculation with *PoPeVYVaRNA-orf1b or PoPeVYVaRNA-orf1ab* did not significantly affect the level of PoPeVYV RNA (Figure 3B). RT-PCR showed that viral RNA was present and had spread systemically in all the inoculated plants, but that mutants of PoPeVYVaRNA did not spread systemically in co-infections with PoPeVYV (Figure 3C). There were leaf rolling symptoms only in plants co-infected with PoPeVYV and PoPeVYVaRNA at 14 dpi (Figure 3C).

### Interveinal Yellowing Symptoms Are Caused by Co-infection With PoPeVYV and PoPeVYV-Associated RNA in *C. frutescens*

**FIGURE 4:**
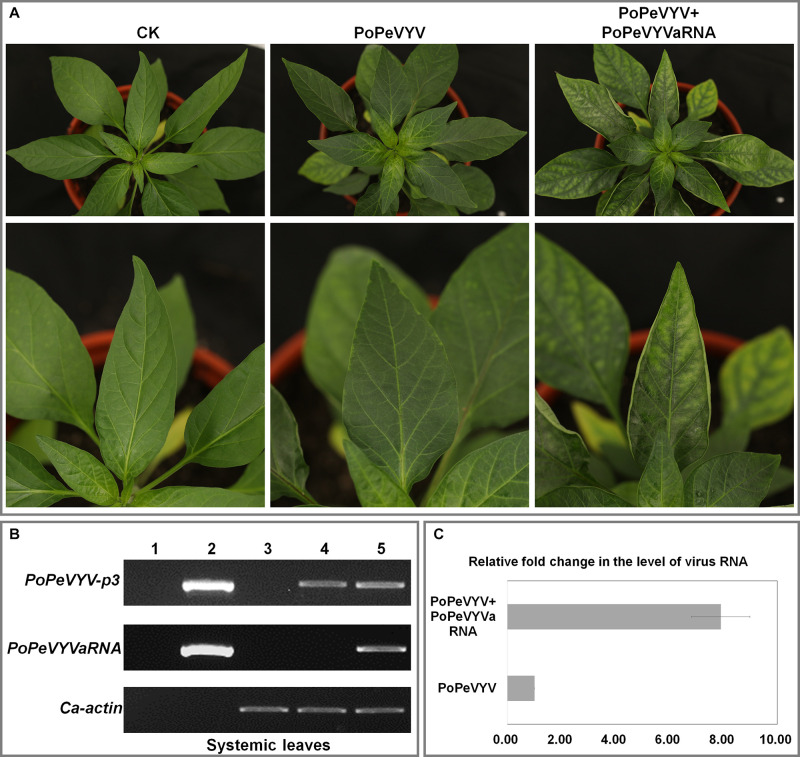
Symptoms caused by PoPeVYV in *Capsicum frutescens* co-infected with PoPeVYVaRNA. **(A)** Phenotype of *C. frutescens* plants infected by aphids with different combinations or empty agrobacterium (CK) 45 days post infiltration. **(B)** RT-PCR confirming the presence of viral RNAs in systemic leaves of infected plants. 1, Negative control; 2, positive control; 3, CK; 4, PoPeVYV; 5, PoPeVYV + PoPeVYVaRNA. **(C)** Relative fold changes of PoPeVYV in systemically infected leaves of *C. frutescens* inoculated with PoPeVYV or PoPeVYV + PoPeVYVaRNA, as shown by quantitative real-time reverse transcription PCR. The means (±*SE*) were calculated from the RNA levels of three individual plants at 45 days post inoculation.

Aphid transmission was used to examine the biological significance of PoPeVYVaRNA in *C. frutescens*. The newly-emerged leaves of plants inoculated with aphids fed only on PoPeVYV virions (SI) had mild interveinal symptoms after 45 days, but when the aphids were fed on a mixture of PoPeVYV and the tlaRNA (MI), the symptoms were much more severe (Figure 4A). RT-PCR showed that the tlaRNA had spread systemically in the MI-treated plants (Figure 4B and Table 2). Quantitative RT-PCR indicated that the level of PoPeVYV RNA in plants transmitted with MI was more than 7.9-fold that in plants transmitted with SI (Figure 4C). RT-PCR indicated that all the aphids fed on SI or MI acquired virus (10/10), but the virus transmission rate by the aphids was very different at, respectively, 17–50 and 67–100% (Table 2).

## Discussion

In this study, we have identified a tombusvirus-like RNA associated with PoPeVYV. tlaRNAs are found in plants co-infected with several viruses in the genus *Polerovirus*. All the tlaRNAs have a very short non-coding region preceding ORF1a at the 5′ end, which encodes a putative product of 25.1–29.3 kDa. Readthrough of the ORF1a amber termination codon allows expression of an 84.6–89.0 kDa protein (ORF1b). The genetic properties of tlaRNAs are similar to viruses in the genus *Umbravirus*, but the conserved polymerase is interrupted by readthrough of the ORF1a amber termination codon instead of slightly overlapping the end (−1 frameshift). Umbraviruses also have a movement protein (MP) that enables them to spread very efficiently within infected plants, but tlaRNAs do not (Ryabov et al., 1998; Campbell et al., 2020). Despite these similarities with umbraviruses and the presence of distinct phylogenetic clades, tlaRNAs have never been formally classified to genera (Lefkowitz et al., 2018; Campbell et al., 2020).

Viral synergism is caused by co-infection of two unrelated viruses, leading to more severe symptoms. Synergistic infection of phloem-restricted poleroviruses and umbraviruses has destructive effects on crop plants and has been well studied (Yoo et al., 2017; Zhou et al., 2017; Yoshida, 2020). Only a few RNAs associated with poleroviruses have been reported, and synergism between them has often been overlooked in the past. It has been shown that several tlaRNAs stimulate the titers of the poleroviruses and enhance the disease symptoms in plants co-infected with their respective poleroviruses (Sanger et al., 1994; Mo et al., 2015; Yoshida, 2020). In this study, we have now also shown synergism between PoPeVYV and its associated RNA (PoPeVYVaRNA) with increased viral titers and symptom severity consistent with field observations (Figures 2, 3 and Supplementary Figure 1). Earlier studies showed that TBTDaRNA could be detected by RT-PCR in 16 of 17 TBTD-affected samples collected from different locations in Yunnan province, showing that TBTDaRNA is a normal component of the tobacco bushy top complex in China (Mo et al., 2011). However, PoPeVYVaRNA was detected by RT-PCR in only eight of 16 PoPeVYV-infected samples, and tlaRNAs do not appear to be essential components of the infections by other PeVYV complexes in fields in Wenshan city (data not shown). This apparent difference between tlaRNAs in their biological effects needs to be examined further.

The plants from the fields described here were infected with various viruses (PeVYVs, ChiVMV, ChiRSV, CMV, etc.), and the severe viral symptoms of PeVYD in the field may therefore be a complicated synergistic effect of mixed infection (Cheng et al., 2011; Laprom et al., 2019; Zhao et al., 2021). The results of this study indicate that one factor affecting PeVYD symptoms is likely to be the co-infection of PoPeVYV and PoPeVYVaRNA.

In conclusion, PoPeVYVaRNA is an associated RNA that depends upon co-infection and encapsidation with PoPeVYV for its systemic movement.

## Data Availability Statement

The datasets presented in this study can be found in online repositories. The names of the repository/repositories and accession number(s) can be found in the article/Supplementary Material.

## Author Contributions

XM, JP, and FY conceived and designed the experiments. HZ, QW, SZ, and YLiu collected the samples. SB, KZ, and MH performed the experiments. YLu and JC analyzed the data. JP, SB, XM, and FY wrote the manuscript. All authors read and approved the final manuscript.

## Conflict of Interest

The authors declare that the research was conducted in the absence of any commercial or financial relationships that could be construed as a potential conflict of interest.

## Footnotes

1 https://talk.ictvonline.org/files/master-species-lists/m/msl/9601

## Supplementary Material

Supplementary Figure 1 Symptoms of virus-infected pod peppers from the field.

Supplementary Figure 2 The deduced amino acid sequences of ORF1b with eight characteristic motifs of + ssRNA virus RdRps in Group II.

Supplementary Table 1 Primers used in this study.

Supplementary Table 2 Accession numbers of tombusvirus-like associated RNAs used for phylogenetic analysis.

## References

[B1] Campbell A. J. Erickson A. Pellerin E. Salem N. Mo X. Falk B. W. (2020). Phylogenetic classification of a group of self-replicating RNAs that are common in co-infections with poleroviruses. *Virus Res.* 276:197831. 10.1016/j.virusres.2019.197831 31790776

[B2] Cheng Y. H. Deng T. C. Chen C. C. Liao J. Y. Chang C. A. Chiang C. H. (2011). First report of pepper mottle virus in bell pepper in Taiwan. *Plant Dis.* 95:617. 10.1094/pdis-10-10-0721 30731963

[B3] Dombrovsky A. Glanz E. Lachman O. Sela N. Doron-Faigenboim A. Antignus Y. (2013). The complete genomic sequence of pepper yellow leaf curl virus (PYLCV) and its implications for our understanding of evolution dynamics in the genus polerovirus. *PLoS One* 8:e70722. 10.1371/journal.pone.0070722 23936244 PMC3728342

[B4] Kamer G. Argos P. (1984). Primary structural comparison of RNA-dependent polymerases from plant, animal and bacterial viruses. *Nucleic Acids Res.* 12 7269–7282. 10.1093/nar/12.18.7269 6207485 PMC320156

[B5] Kamran A. Lotos L. Amer M. A. Al-Saleh M. A. Alshahwan I. M. Shakeel M. T. (2018). Characterization of pepper leafroll chlorosis virus, a new polerovirus causing yellowing disease of bell pepper in Saudi Arabia. *Plant Dis.* 102 318–326. 10.1094/pdis-03-17-0418-re 30673532

[B6] Knierim D. Tsai W. S. Kenyon L. (2013). Analysis of sequences from field samples reveals the presence of the recently described pepper vein yellows virus (genus Polerovirus) in six additional countries. *Arch. Virol.* 158 1337–1341. 10.1007/s00705-012-1598-y 23307365

[B7] Koonin E. V. (1991). The phylogeny of RNA-dependent RNA polymerases of positive-strand RNA viruses. *J. Gen. Virol.* 72 2197–2206. 10.1099/0022-1317-72-9-2197 1895057

[B8] Kumar S. Stecher G. Li M. Knyaz C. Tamura K. (2018). MEGA X: molecular evolutionary genetics analysis across computing platforms. *Mol. Biol. Evol.* 35 1547–1549. 10.1093/molbev/msy096 29722887 PMC5967553

[B9] Laprom A. Nilthong S. Chukeatirote E. (2019). Incidence of viruses infecting pepper in Thailand. *Biomol. Concepts* 10 184–193. 10.1515/bmc-2019-0021 31743101

[B10] Le S. Q. Gascuel O. (2008). An improved general amino acid replacement matrix. *Mol. Biol. Evol.* 25 1307–1320. 10.1093/molbev/msn067 18367465

[B11] Lefkowitz E. J. Dempsey D. M. Hendrickson R. C. Orton R. J. Siddell S. G. Smith D. B. (2018). Virus taxonomy: the database of the International Committee on Taxonomy of Viruses (ICTV). *Nucleic Acids Res.* 46 D708–D717. 10.1093/nar/gkx932 29040670 PMC5753373

[B12] Liu M. Liu X. Li X. Zhang D. Dai L. Tang Q. (2016). Complete genome sequence of a Chinese isolate of pepper vein yellows virus and evolutionary analysis based on the CP, MP and RdRp coding regions. *Arch. Virol.* 161 677–683. 10.1007/s00705-015-2691-9 26620586

[B13] Lotos L. Olmos A. Orfanidou C. Efthimiou K. Avgelis A. Katis N. I. (2017). Insights into the etiology of polerovirus-induced pepper yellows disease. *Phytopathology* 107 1567–1576. 10.1094/PHYTO-07-16-0254-R 28786341

[B14] Maina S. Edwards O. R. Jones R. A. (2016). First complete genome sequence of pepper vein yellows virus from Australia. *Genome Announc.* 4 450–416. 10.1128/genomeA.00450-16 27231375 PMC4882956

[B15] Mo X. H. Chen Z. B. Chen J. P. (2010). Complete nucleotide sequence and genome organization of a Chinese isolate of tobacco vein distorting virus. *Virus Genes* 41 425–431. 10.1007/s11262-010-0524-1 20740310

[B16] Mo X. H. Chen Z. B. Chen J. P. (2011). Molecular identification and phylogenetic analysis of a viral RNA associated with the Chinese tobacco bushy top disease complex. *Ann. Appl. Biol.* 158 188–193. 10.1111/j.1744-7348.2010.00452.x

[B17] Mo X. H. Xu P. Zhao X. N. Zhang L. F. Qin X.Y. Xia Z. Y. (2015). “The interactions between tobacco vein distorting virus and tobacco bushy top disease-associated RNA,” in *Proceedings of the 2015 CORESTA Meeting, Agronomy/Phytopathology* (Izmir: CORESTA). Available online at: https://www.coresta.org/abstracts/interactions-between-tobacco-vein-distorting-virus-and-tobacco-bushy-top-disease

[B18] Murakami R. Nakashima N. Hinomoto N. Kawano S. Toyosato T. (2011). The genome sequence of pepper vein yellows virus (family Luteoviridae, genus Polerovirus). *Arch. Virol.* 156 921–923. 10.1007/s00705-011-0956-5 21400195 PMC3081434

[B19] Ryabov E. V. Oparka K. J. Santa Cruz S. Robinson D. J. Taliansky M. E. (1998). Intracellular location of two groundnut rosette umbravirus proteins delivered by PVX and TMV vectors. *Virology* 242 303–313. 10.1006/viro.1997.9025 9514976

[B20] Sanger M. Passmore B. Falk B. W. Bruening G. Ding B. Lucas W. J. (1994). Symptom severity of beet western yellows virus-strain ST9 is comferred by the ST9-associated RNA and is not associated with virus release from the phloem. *Virology* 200 48–55. 10.1006/viro.1994.1161 8128637

[B21] Syller J. (2002). Umbraviruses: The unique plant viruses that do not encode a capsid protein. *Acta Microbiol. Polon.* 51 99–113. 12363080

[B22] Yoo R. H. Lee S. W. Lim S. Zhao F. Igori D. Baek D. (2017). Complete genome analysis of a novel umbravirus-polerovirus combination isolated from Ixeridium dentatum. *Arch. Virol.* 162 3893–3897. 10.1007/s00705-017-3512-0 28905257

[B23] Yoshida N. (2020). Biological and genetic characterization of carrot red leaf virus and its associated virus/RNA isolated from carrots in Hokkaido, Japan. *Plant Pathol.* 69 1379–1389. 10.1111/ppa.13202

[B24] Zhao K. Yin Y. Hua M. Wang S. Mo X. Yuan E. (2021). Pod pepper vein yellows virus, a new recombinant polerovirus infecting Capsicum frutescens in Yunnan province, China. *Virol. J.* 18:42. 10.1186/s12985-021-01511-5 33622354 PMC7901092

[B25] Zhou C. J. Zhang X. Y. Liu S. Y. Wang Y. Li D. W. Yu J. L. (2017). Synergistic infection of BrYV and PEMV 2 increases the accumulations of both BrYV and BrYV-derived siRNAs in Nicotiana benthamiana. *Sci. Rep.* 7:45132. 10.1038/srep45132 28345652 PMC5366869

