# Supplementary figures and images for: Biological and Genetic Characterization of Pod Pepper Vein Yellows Virus-Associated RNA From Capsicum frutescens in Wenshan, China
Source: Front Microbiol. 2021 Apr 15;12:662352. doi: 10.3389/fmicb.2021.662352 (PMC8083956; doi:10.3389/fmicb.2021.662352)

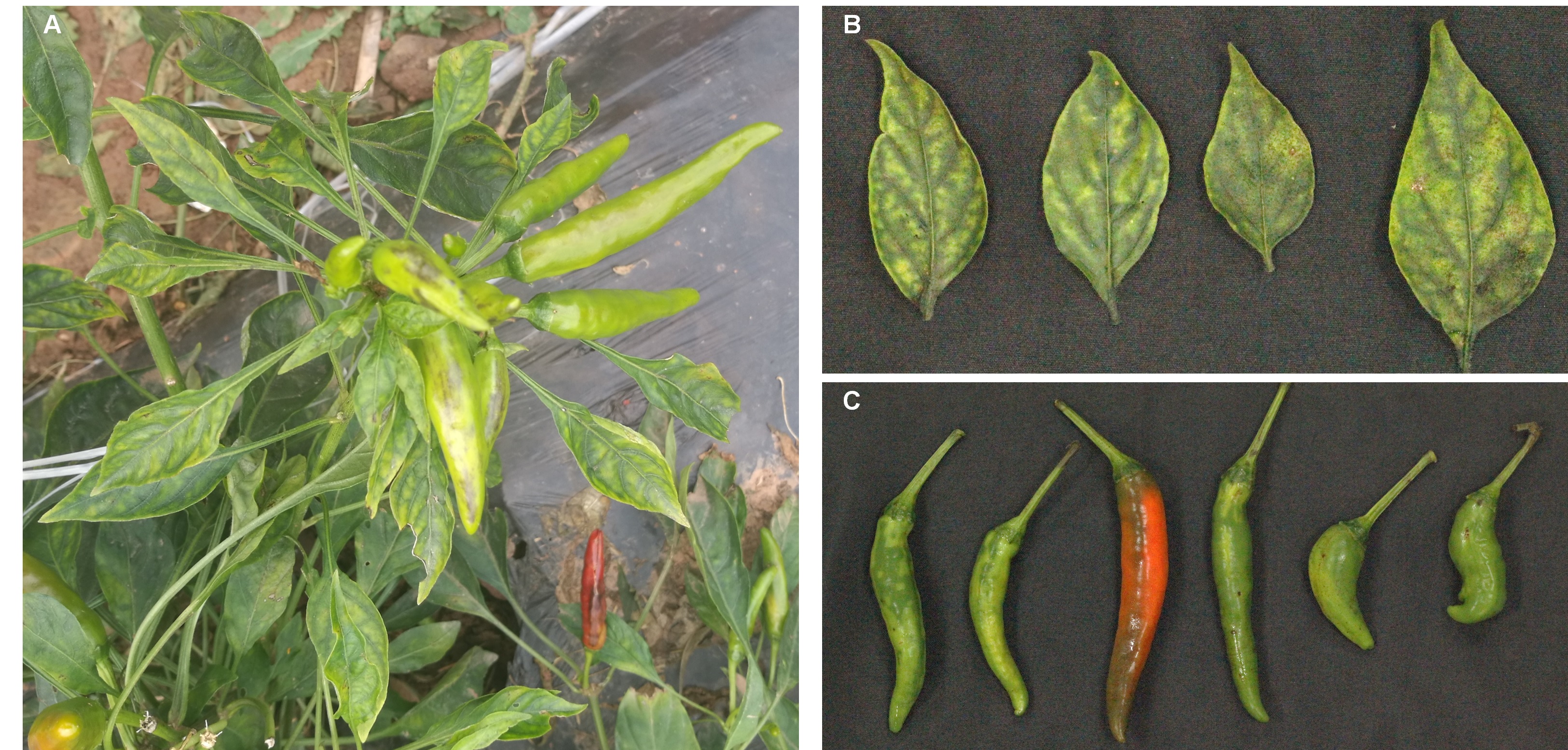

Supplement: Supplementary Figure 1 — Symptoms of virus-infected pod peppers from the field. [file Image_1.JPEG]

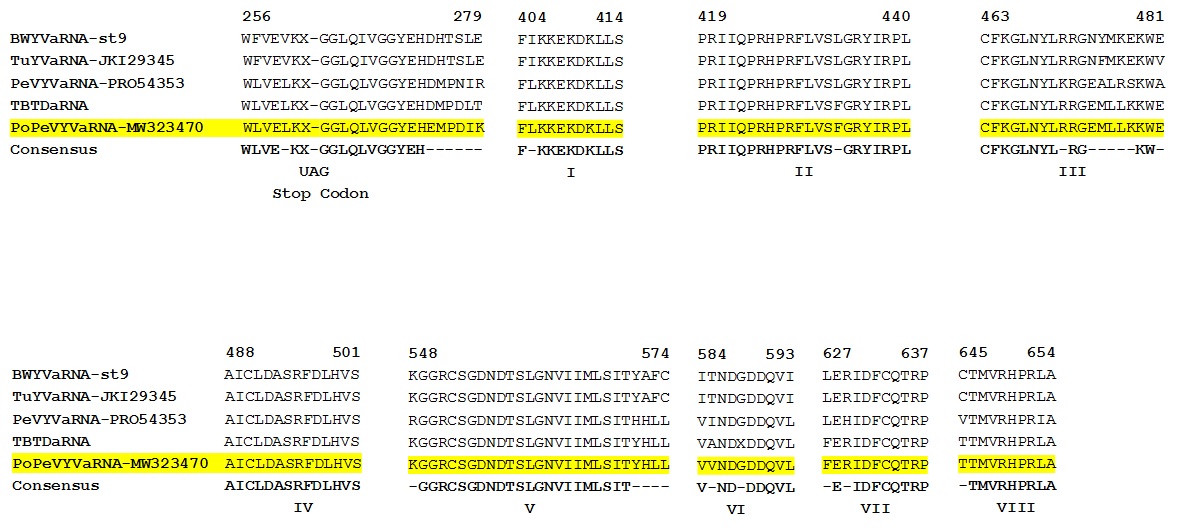

Supplement: Supplementary Figure 2 — The deduced amino acid sequences of ORF1b with eight characteristic motifs of + ssRNA virus RdRps in Group II. [file Image_2.JPEG]
