# Supplementary material for: Biological and Genetic Characterization of Pod Pepper Vein Yellows Virus-Associated RNA From Capsicum frutescens in Wenshan, China
Source: Front Microbiol. 2021 Apr 15;12:662352. doi: 10.3389/fmicb.2021.662352 (PMC8083956; doi:10.3389/fmicb.2021.662352)
Supplement: Supplementary Table 1 — Primers used in this study. [file Table_1.DOCX]

Table S1. Primers used in this study

| Objective | name | sequence |
| --- | --- | --- |
| Sequence validation | AR3F | 5′-CAGATGAATTTCTGGCGTGCTA-3′ |
|  | AR5R | 5′-TCCACCATCAACACYTGRTCRTCTCCRTCRTT-3′ |
|  | tlaRNA-WS f | 5′-GGGGATTCATGGAGCACACAAGAGG-3′ |
|  | tlaRNA-WS r | 5′-GGGGCGGTGTACCTGGTAAACAAAGTG-3′ |
| RACE PCR | M4 | 5′-GTTTTCCCAGTCACGAC-3′ |
|  | M4T | 5'-GTTTTCCCAGTCACGAC(T)_15_-3′ |
|  | ZHM2 | 5'-gAggAgggAggggAAgAg-3′ |
|  | ZHM1 | 5'-PO_4_-CTCTTCCCCTCCCTCCTC-NH_2_-3′ |
|  | 3' -RACE-F | 5'- GCGTAGAGATAGATTAGGACCG -3′ |
|  | 5'-RACE-R | 5'- TCTTCCGAATAGCCAACC -3′ |
| RT-PCR or qRT-PCR detection | Nb-UBC f | 5′-TTTCGGTCCTGATGATACTCCC-3′ |
|  | Nb-UBC r | 5′-CACAGAGCAAAGACTGGATTGA-3′ |
|  | Ca-actin f | 5′- CAGCCTCTTGTCTGTGATAATG -3′ |
|  | Ca-actin r | 5′- GAGCATAACCTTCATAGATGGG -3′ |
|  | RT-tla f | 5′-GACTTCACTGTTCCAGCGATTC-3′ |
|  | RT-tla r | 5′-TCATCTCCGTCATTCACGAC-3′ |
|  | qPoPeVYV-P3 f | 5′-TAGTCTCCAATCCACCCTGC-3′ |
|  | qPoPeVYV-P3 r | 5′-TAACCCATCACTCCTCCCAC |
| Colony PCR | Inf-tlaRNA f | 5′- AAGTTCATTTCATTTGGAGAGGGGGGATTCATGGAGCACACAAG-3′ |
|  | Inf-tlaRNA r | 5′-GGAGATGCCATGCCGACCCGGGGCGGTGTACCTGGTAAAC-3′ |
| Linearize vector | Vec-pCB301 f | 5'-GGGTCGGCATGGCATCTCCA-3′ |
|  | Vec-pCB301 r | 5'-CCTCTCCAAATGAAATGAACTT-3′ |

1. The Sequence validation primers were used to verify the virus sequences.
2. The RACE PCR primers were used to obtain the complete terminal sequences of PoPeVYV.
3. The PCR detection primers were used to detect the virus.
4. The qRT-PCR detection primers were used to identify the viral RNA level.
5. The Colony PCR and Linearize vector primers were used to generate infectious clones (pCB-PoPeVYVaRNA).
