# Supplementary material for: Biological and Genetic Characterization of Pod Pepper Vein Yellows Virus-Associated RNA From Capsicum frutescens in Wenshan, China
Source: Front Microbiol. 2021 Apr 15;12:662352. doi: 10.3389/fmicb.2021.662352 (PMC8083956; doi:10.3389/fmicb.2021.662352)
Supplement: Supplementary Table 2 — Accession numbers of tombusvirus-like associated RNAs used for phylogenetic analysis. [file Table_2.DOCX]

Table S2. Accession numbers of tombusvirus-like associated RNAs used for phylogenetic analysis

| **TlaRNA abbreviations** | **Name** | **Acronym** | **Isolate** | **Accession^a^** | **Country** |
| --- | --- | --- | --- | --- | --- |
| CRLVaRNA-HK2 | carrot red leaf virus associated RNA | CRLVaRNA | HK2 | LC434070 | Japan |
| CRLVaRNA-SH |  |  | SH | LC434069 | Japan |
| CRLVaRNA-alpha |  |  | alpha | KM486095 | USA |
| CRLVaRNA-beta |  |  | beta | KM486096 | USA |
| CRLVaRNA-gamma |  |  | Gamma | KM486092 | USA |
| CRLVaRNA-sigma |  |  | Sigma | KM486093 | USA |
| CRLVaRNA-a8 |  |  | a8 | AF020616 | USA |
| CRLVaRNA-a25 |  |  | a25 | AF020617 | USA |
| TVDVaRNA | tobacco bushy top disease-associated RNA | TBTDaRNA | TBTDaRNA | EF529625 | China |
| TuYVaRNA-JKI29345 | turnip yellows virus associated RNA | TuYVaRNA | JKI 29345 | MK450521 | Germany |
| CABYVaRNA-CABYV | cucurbit aphid borne yellows virus associated RNA | CABYVaRNA | CABYV | KM486094 | USA |
| BWYVaRNA-st9 | beet western yellows st9 associated RNA | BWYVaRNA | ST9 | L04281 | USA |
| PoPeVYVaRNA | pod pepper vein yellows virus associated RNA | PoPeVYVaRNA | Wenshan | MW323470* | China |
| PeVYVaRNA-PRO54353 | pepper vein yellows virus associated RNA | PeVYVaRNA | PRO54353 | MT321510 | Netherlands |

^a^ Sequences determined in this study are indicated with an asterisk.
